# New risk factors and new tendency for central nervous system relapse in patients with diffuse large B-cell lymphoma: a retrospective study

**DOI:** 10.1186/s40880-016-0150-y

**Published:** 2016-09-13

**Authors:** Qing-Qing Cai, Li-Yang Hu, Qi-Rong Geng, Jie Chen, Zhen-Hai Lu, Hui-Lan Rao, Qing Liu, Wen-Qi Jiang, Hui-Qiang Huang, Tong-Yu Lin, Zhong-Jun Xia

**Affiliations:** 1 State Key Laboratory of Oncology in South China, Collaborative Innovation Center for Cancer Medicine, Sun Yat-sen University Cancer Center, Guangzhou, 510060 Guangdong P. R. China; 2 Department of Medical Oncology, Sun Yat-sen University Cancer Center, 651 Dongfeng Road East, Guangzhou, 510060 Guangdong P. R. China; 3 Department of Hematology Oncology, Sun Yat-sen University Cancer Center, Guangzhou, 510060 Guangdong P. R. China; 4 Guangdong Province Key Laboratory of Arrhythmia and Electrophysiology, Guangzhou, 510120 Guangdong P. R. China; 5 Department of Radiotherapy, Sun Yat-sen Memorial Hospital of Sun Yat-sen University, Guangzhou, 510120 Guangdong P. R. China; 6 Department of Colorectal Carcinoma, Sun Yat-sen University Cancer Center, Guangzhou, 510060 Guangdong P. R. China; 7 Department of Pathology, Sun Yat-sen University Cancer Center, Guangzhou, 510060 Guangdong P. R. China; 8 Department of Cancer Prevention Research, Sun Yat-sen University Cancer Center, Guangzhou, 510060 Guangdong P. R. China

**Keywords:** Diffuse large B-cell lymphoma, Central nervous system relapse, Risk factor, Rituximab, Intrathecal chemotherapy prophylaxis

## Abstract

**Background:**

In patients with diffuse large B-cell lymphoma (DLBCL), central nervous system (CNS) relapse is uncommon but is nearly always fatal. This study aimed to determine the risk factors for CNS relapse in DLBCL patients and to evaluate the efficacy of rituximab and intrathecal chemotherapy prophylaxis for CNS relapse reduction.

**Methods:**

A total of 511 patients with newly diagnosed DLBCL treated at the Sun Yat-sen University Cancer Center between January 2003 and December 2012 were included in the study. Among these patients, 376 received R-CHOP regimen (rituximab, cyclophosphamide, doxorubicin, vincristine, and prednisone) as primary treatment, and 135 received CHOP regimen (cyclophosphamide, doxorubicin, vincristine, and prednisone) as primary treatment. Intrathecal chemotherapy prophylaxis (methotrexate plus cytarabine) was administered to those who were deemed at high risk for CNS relapse. In the entire cohort and in the R-CHOP set in particular, the Kaplan–Meier method coupled with the log-rank test was used for univariate analysis, and the Cox proportional hazards model was used for multivariate analysis. Differences were evaluated using a two-tailed test, and *P* < 0.05 was considered significant.

**Results:**

At a median follow-up of 46 months, 25 (4.9%) patients experienced CNS relapse. There was a trend of reduced occurrence of CNS relapse in patients treated with rituximab; the 3-year cumulative CNS relapse rates were 7.1% in CHOP group and 2.7% in R-CHOP group (*P* = 0.045). Intrathecal chemotherapy prophylaxis did not confer much benefit in terms of preventing CNS relapse. Bone involvement [hazard ratio (HR) = 4.21, 95% confidence interval (CI) 1.38–12.77], renal involvement (HR = 3.85, 95% CI 1.05–14.19), alkaline phosphatase (ALP) >110 U/L (HR = 3.59, 95% CI 1.25–10.34), serum albumin (ALB) <35 g/L (HR = 3.63, 95% CI 1.25–10.51), treatment with rituximab (HR = 0.34, 95% CI 0.12–0.96), and a time to complete remission ≤ 108 days (HR = 0.22, 95% CI 0.06–0.78) were independent predictive factors for CNS relapse in the entire cohort. Bone involvement (HR = 4.44, 95% CI 1.08–18.35), bone marrow involvement (HR = 11.70, 95% CI 2.24–60.99), and renal involvement (HR = 10.83, 95% CI 2.27–51.65) were independent risk factors for CNS relapse in the R-CHOP set.

**Conclusions:**

In the present study, rituximab decreased the CNS relapse rate of DLBCL, whereas intrathecal chemotherapy prophylaxis alone was not sufficient for preventing CNS relapse. Serum levels of ALB and ALP, and the time to complete remission were new independent predictive factors for CNS relapse in the patients with DLBCL. In the patients received R-CHOP regimen, a trend of increased CNS relapse was found to be associated with extranodal lesions.

## Background

Diffuse large B-cell lymphoma (DLBCL) is the most common lymphoid malignancy in adults, comprising 30%–35% of all non-Hodgkin lymphoma (NHL) cases [1, 2]. According to recent estimates, patients with DLBCL have a 5% overall risk of central nervous system (CNS) relapse [3]. Although relatively uncommon, CNS relapse is nearly always fatal. This CNS event can dramatically shorten the overall survival (OS) of DLBCL patients to less than 6 months [4]. Early detection of high-risk cases and effective CNS prophylaxis or treatment are the main strategies for outcome improvement. Several studies have identified high-risk patients to be those with advanced stages of disease, elevated lactate dehydrogenase (LDH) level, and involvement of multiple extranodal sites or specific extranodal sites (e.g. the paranasal sinus, breast, testicle, bone marrow, kidney, orbital and epidural spaces) [5, 6]. *MYC* gene rearrangements was also reported to be a risk factor for DLBCL, and treatment regimens similar to those for Burkitt lymphoma were recommended to DLBCL patients [7, 8]. The National Comprehensive Cancer Network (NCCN) guideline recommends using the international prognostic index (IPI) and measuring renal involvement to evaluate the risk of CNS relapse in DLBCL patients. However, even knowing the risk factors and using this screening method, only half of high-risk patients can be identified [9]. In the era of rituximab, high-risk cases should be effectively evaluated for early intervention. Whether other risk factors can predict CNS relapse remains an open question.

The addition of the chimeric anti-CD20 monoclonal antibody rituximab to CHOP regimen (cyclophosphamide, doxorubicin, vincristine, and prednisone), termed the R-CHOP regimen, has greatly improved the survival of DLBCL patients [10–15]. Currently, chemo-immunotherapy with R-CHOP regimen or its derivatives is the standard first-line therapy for DLBCL [11, 13, 16]. Because rituximab can hardly penetrate the blood–brain barrier to reach the CNS, its efficacy in preventing CNS relapse is still controversial [17]. To cross the blood–brain barrier and increase therapeutic concentrations in the brain and cerebrospinal fluid (CSF), intrathecal (IT) administration of methotrexate (MTX) or cytarabine (Ara-C) is a simple and well-accepted method for CNS prophylaxis. Nonetheless, the benefit of IT chemotherapy administration for CNS relapse prophylaxis has been questioned in recent years. Certain studies have suggested that IT chemotherapy prophylaxis alone is an inadequate strategy for the prevention of CNS relapse [4, 6, 18, 19].

In this study in the post-rituximab era, we aimed to retrospectively explore the risk factors for CNS relapse in an entire cohort and in an R-CHOP set in particular and to evaluate the efficacy of rituximab and IT chemotherapy prophylaxis for CNS relapse reduction.

## Methods

### Patient selection

Patients with newly diagnosed DLBCL treated at the Sun Yat-sen University Cancer Center between January 2003 and December 2012 were included in this retrospective study. All of them had been diagnosed by biopsy according to the 2001 or 2008 World Health Organization classification. The patients in this study were 18 years or older and lacked CNS relapse at the time of diagnosis. Other selection criteria included receiving anthracycline-based chemotherapy as a first-line treatment with curative intent; lacking human immunodeficiency virus (HIV) infection; and having adequate clinical information available, including follow-up data. The Ann-Arbor staging system and the IPI were used for staging evaluation and risk stratification. This study was approved by the Institutional Review Board of Sun Yat-sen University Cancer Center (Approval No. YB2014-11-20).

### Treatment and IT chemotherapy prophylaxis

R-CHOP regimen was recommended to all enrolled patients as the standard first-line treatment. However, several patients chose chemotherapy without rituximab. The entire cohort could be divided into R-CHOP and CHOP groups. R-CHOP/CHOP-based regimens with minor modifications were also regarded as R-CHOP/CHOP chemotherapy.

IT chemotherapy prophylaxis was administered to those patients who were deemed at high risk of CNS relapse at the discretion of the physician. In general, IT chemotherapy prophylaxis was performed more often in patients with bulky mass; a high level of ki-67; and involvement of the testis, breast, or kidney in our cancer center. The regimen consisted of 15 mg MTX and 50 mg Ara-C administered by lumbar puncture on the first day of each cycle. CSF samples from these patients were sent for laboratory tests. Diagnosis of CNS relapse was based on radiologic evidence, cytological findings, or clinical symptoms of CNS relapse.

### Follow-up

The number of first-line chemotherapy cycles and subsequent treatment were decided according to the stage of disease, the response to previous treatment, and the patients’ characteristics. After treatment, patients were followed up every 3 months in the first 3 years and every 6 months thereafter. During the follow-up period, routine examinations included physical examination, standard laboratory tests, echocardiography, and a whole-body computed tomography (CT) scan or fluorodeoxyglucose-positron emission tomography (FDG-PET). Lumbar puncture, head CT, or head magnetic resonance imaging (MRI) was mainly administered to those with the clinical symptoms of CNS relapse. The final follow-up date in this study was May 31, 2015.

### Statistical analysis

The clinical characteristics of the entire cohort and the patients who experienced CNS relapse in the R-CHOP and CHOP sets were compared using the Chi square test or Fisher’s exact test.

CNS relapse was the primary endpoint of our study. The CNS relapse-free survival rate was calculated from the date of initial diagnosis to the date of CNS relapse, death without CNS relapse, or last follow-up. The Kaplan–Meier method coupled with the log-rank test was used for univariate analyses and generation of survival curves. All factors with *P* values less than 0.10 were included in the multivariate analysis using the Cox proportional hazards model. The secondary endpoints were OS and progression-free survival (PFS). OS was defined as the time from the date of diagnosis to the date of death or last follow-up. PFS was calculated from the date of diagnosis to the date of disease progression/relapse, death, or last follow-up. The OS and PFS of patients with or without CNS relapse were estimated using the Kaplan–Meier method and the log-rank test.

The time to complete remission (TTC) was calculated from the date of first treatment to the date of first complete remission (CR). Receiver operating characteristic (ROC) curve analysis was used to determine the optimal cutoff points for the TTC. The TTC was also included in the univariate and multivariate analyses. Differences were evaluated using a two-tailed test, and *P* < 0.05 was considered statistically significant. All statistical analysis was carried out using SPSS version 19.0 (IBM, Armonk, NY, USA).

## Results

### Characteristics of patients

**Fig. 1:**
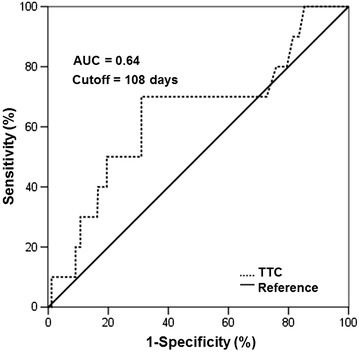
Receiver operating characteristic (ROC) curve analysis detected the cutoff point of time to complete remission (TTC) as 108 days. *AUC* area under curve

A total of 511 patients with DLBCL were included in our analysis. Among these patients, 295 (57.7%) were men, and 216 (42.3%) were women. The median age was 52 years old (range, 18–82 years). There were 376 (73.6%) patients who received R-CHOP regimen as primary treatment, and the remaining 135 (26.4%) patients received CHOP regimen; 217 (42.5%) with advanced-stage (stages III and IV) disease; involvement of more than one extranodal organ was found in 94 (18.4%) patients, and an elevated serum LDH level was found in 214 (41.9%) patients. CNS prophylaxis with IT MTX and Ara-C was administered in 62 (12.1%) patients; the median number of IT chemotherapy prophylaxis cycles was four (range 1–8). As recommended by the NCCN model for CNS relapse, the 16 patients who scored 4–6 were recognized as high-risk cases, of whom four received IT chemotherapy prophylaxis. At a median follow-up of 46 months (range 1–129 months), 25 (4.9%) patients experienced CNS relapse; only five among them were in the high-risk group based on the NCCN model. The median time to CNS relapse among the 25 patients was 7 months (range 1–46 months). The TTC was calculated in 396 (77.5%) patients, who achieved CR at least once. ROC curve analysis revealed the optimal cutoff value of TTC as 108 days, with an area under the curve of 0.64 (Fig. 1). Among the patients who achieved CR, 12 received rituximab as maintenance therapy. The detailed baseline characteristics of the entire cohort and the CNS relapse cohort are displayed in Table 1. There was no significant difference in clinical characteristics between the R-CHOP and the CHOP sets in the two cohorts, except for a difference in the TTC. Compared with the CHOP set, more patients in the R-CHOP set achieved a rapid CR in both cohorts.

**Table 1:** Characteristics of the 511 diffuse large B-cell lymphoma (DLBCL) patients in the entire cohort and the central nervous system (CNS) relapse cohort (CHOP vs. R-CHOP)

Clinical factor	Entire cohort [cases (%)]			CNS relapse cohort [cases (%)]		
	CHOP	R-CHOP	*P*	CHOP	R-CHOP	*P*
Total	135	376		11	14	
Age			0.563			1.000
≤60 years	98 (72.6)	263 (69.9)		8 (72.7)	10 (71.4)	
>60 years	37 (27.4)	113 (30.1)		3 (27.3)	4 (28.6)	
Gender			0.694			0.115
Male	76 (56.3)	219 (58.2)		5 (45.5)	11 (78.6)	
Female	59 (43.7)	157 (41.8)		6 (54.5)	3 (21.4)	
Stage			0.892			0.434
I or II	77 (57.0)	217 (57.7)		5 (45.5)	4 (28.6)	
III or IV	58 (43.0)	159 (42.3)		6 (54.5)	10 (71.4)	
B symptoms			0.663			1.000
No	95 (70.4)	272 (72.3)		7 (63.6)	8 (57.1)	
Yes	40 (29.6)	104 (27.7)		4 (36.4)	6 (42.9)	
Bulky disease^a,b^			0.238			0.500
No	91 (67.4)	252 (67.0)		6 (54.5)	8 (57.1)	
Yes	10 (7.4)	17 (4.5)		0 (0)	2 (14.3)	
Involved extranodal sites^c^			0.617			0.414
≤1	112 (83.0)	303 (80.6)		8 (72.7)	7 (50)	
>1	23 (17.0)	71 (18.9)		3 (27.3)	7 (50)	
Sinus involvement			0.971			0.604
No	119 (88.1)	331 (88.0)		10 (90.9)	11 (78.6)	
Yes	16 (11.9)	45 (12.0)		1 (9.1)	3 (21.4)	
Bone involvement			0.947			0.407
No	120 (88.9)	335 (89.1)		9 (81.8)	9 (64.3)	
Yes	15 (11.1)	41 (10.9)		2 (18.2)	5 (35.7)	
Bone marrow involvement			0.177			0.230
No	133 (98.5)	360 (95.7)		11 (100)	11 (78.6)	
Yes	2 (1.5)	16 (4.3)		0 (0)	3 (21.4)	
Liver involvement			0.792			0.487
No	131 (97.0)	361 (96.0)		11 (100)	12 (85.7)	
Yes	4 (3.0)	15 (4.0)		0 (0)	2 (14.3)	
Testicular involvement			0.526			1.000
No	131 (97.0)	368 (97.9)		11 (100)	13 (92.9)	
Yes	4 (3.0)	8 (2.1)		0 (0)	1 (7.1)	
Renal involvement			0.782			0.661
No	130 (96.3)	360 (95.7)		9 (81.8)	10 (71.4)	
Yes	5 (3.7)	16 (4.3)		2 (18.2)	4 (28.6)	
Breast involvement			0.318			–
No	130 (96.3)	368 (97.9)		11 (100)	14 (100)	
Yes	5 (3.7)	8 (2.1)		0 (0)	0 (0)	
Female genital tract involvement			0.063			–
No	131 (97.0)	373 (99.2)		11 (100)	14 (100)	
Yes	4 (3.0)	3 (0.8)		0 (0)	0 (0)	
IPI			0.096			0.208
0–2	113 (83.7)	289 (76.9)		9 (81.8)	7 (50.0)	
3–5	22 (16.3)	87 (23.1)		2 (18.2)	7 (50.0)	
LDH^d^			0.768			0.677
≤1 ULN^e^	79 (58.5)	215 (57.2)		3 (27.3)	6 (42.9)	
>1 ULN	55 (40.7)	159 (42.3)		8 (72.7)	8 (57.1)	
ALB			0.464			0.656
<35 g/L	114 (84.4)	327 (87.0)		4 (36.4)	3 (21.4)	
≥35 g/L	21 (15.6)	49 (13.0)		7 (63.6)	11 (78.6)	
ALP^f^			0.529			1.000
≤110 U/L	119 (88.1)	341 (90.7)		8 (72.7)	10 (71.4)	
>110 U/L	15 (11.1)	35 (9.3)		3 (27.3)	4 (28.6)	
IT chemotherapy prophylaxis^g^			0.640			0.661
No	117 (86.7)	329 (87.5)		9 (81.8)	10 (71.4)	
Yes	18 (13.3)	44 (11.7)		2 (18.2)	4 (28.6)	
TTC^h^			0.016			0.003
≤108 days	54 (40.0)	207 (55.1)		0 (0)	7 (50.0)	
>108 days	67 (49.6)	155 (41.2)		10 (90.9)	3 (21.4)	

*CHOP* cyclophosphamide, doxorubicin, vincristine, and prednisone, *R-CHOP* rituximab, cyclophosphamide, doxorubicin, vincristine, and prednisone, *IPI* international prognostic index, *LDH* lactate dehydrogenase, *ULN* upper limit of normal, *ALB* albumin, *ALP* alkaline phosphatase, *IT* intrathecal, *TTC* time to complete remission

^a^Bulky disease: mass ≥7.5 cm

^b^In the entire cohort, the data of 34 patients in the CHOP group and seven patients in the R-CHOP group were missing; in the CNS relapse cohort, the data of five patients in the CHOP group and four patients in the R-CHOP group were missing

^c^In the entire cohort, the data of two patients in the R-CHOP group were missing

^d^In the entire cohort, the data of one patient in the CHOP group and two patients in the R-CHOP group were missing

^f^In the entire cohort, the data of one patient in the CHOP group were missing

^g^In the entire cohort, the data of three patients in the R-CHOP group were missing

^h^In the entire cohort, the data of 14 patients in the CHOP group and 14 patients in the R-CHOP group were missing; in the CNS relapse cohort, the data of one patient in the CHOP group and four patients in the R-CHOP group were missing

### CNS relapse and its risk factors in the entire cohort

A total of 11 (8.1%) patients in the CHOP set and 14 (3.7%) in the R-CHOP set developed CNS relapse within the follow-up period (*P* = 0.041); the median time to CNS relapse was 7 months in the CHOP set and 6.5 months in the R-CHOP set. Comparing the two sets, there was a trend of a reduced likelihood of CNS relapse in the R-CHOP set: the 3-year CNS relapse rate was 2.7% in the R-CHOP set and 7.1% in the CHOP set (*P* = 0.045). Univariate analysis of the effect of rituximab maintenance on CNS relapse in patients with CR showed a *P* value of 0.997, implying that rituximab maintenance could not decrease the occurrence of CNS relapse. Among patients treated with IT chemotherapy prophylaxis, 6 (9.7%) experienced CNS relapse, whereas 19 (4.2%) among those without IT chemotherapy prophylaxis experienced CNS relapse. The addition of IT chemotherapy prophylaxis did not confer much benefit for CNS relapse: the 3-year CNS relapse rates in patients with and without IT were 6.5% and 2.0% (*P* = 0.062). In the high-risk group, as recognized by the NCCN model, univariate analysis of the effect of IT chemotherapy prophylaxis on CNS relapse showed a *P* value of 0.171. Therefore, in this small group of high-risk patients, IT chemotherapy prophylaxis also showed no benefit in reducing CNS relapse. Patients who experienced CNS relapse were less likely to achieve a rapid CR than those who did not experience CNS relapse (28.0% vs. 52.3%, *P* = 0.023); patients who achieved their first CR within 108 days had a trend towards a lower risk of CNS relapse than those who failed to reach CR within 108 days (1.1% vs. 7.7%, *P* < 0.001).

**Fig. 2:**
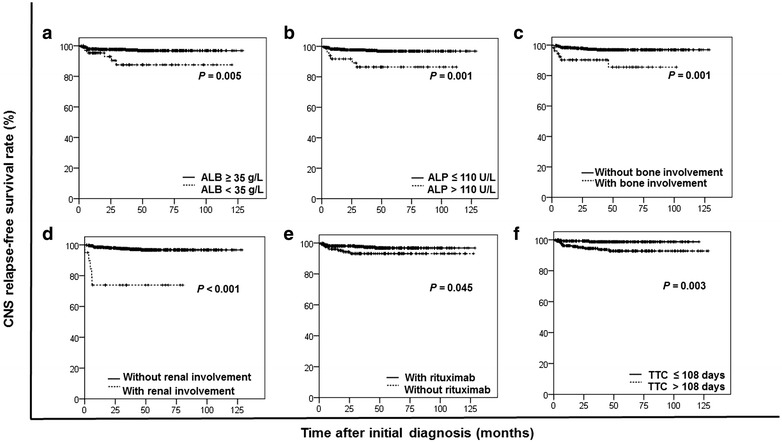
Kaplan–Meier analysis estimated central nervous system (CNS) relapse-free survival for the 511 diffuse large B-cell lymphoma (DLBCL) patients stratified by independent risk factors. A low albumin (ALB) level at diagnosis (**a**), a high alkaline phosphatase (ALP) level (**b**), bone involvement (**c**), renal involvement (**d**), a lack of rituximab treatment (**e**), and a long time to complete remission (TTC) (**f**) were associated with short CNS relapse-free survival

Univariate analysis of the entire cohort detected that the increased risk of CNS relapse was associated with advanced-stage (stage III or IV) disease, an IPI ≥3, involvement of more than one extranodal site, involvement of the bone marrow, involvement of the bone, involvement of the kidney, an absolute lymphocyte count <1.3 × 10^9^/L, elevated serum LDH level (>245 U/L), elevated alkaline phosphatase (ALP) level (>110 U/L), reduced serum albumin (ALB) level (<35 g/L), reduced high-density lipoprotein (HDL) level (<0.78 mmol/L), reduced serum apolipoprotein-A1 (Apo-A1) level (<1.05 g/L), lack of rituximab treatment, failure to attain CR after first-line therapy, and a TTC >108 days (all *P* < 0.05). Additional risk factors with *P* values less than 0.10 were also identified: an absolute white blood cell (WBC) count <4.0 × 10^9^/L, a platelet count ≥100 × 10^9^/L, serum total protein (TP) <60 g/L, and receipt of IT chemotherapy prophylaxis. The results of the univariate analysis are listed in Table 2, and the CNS relapse-free survival curves of patients with independent risk factors can be observed in Fig. 2.

**Table 2:** Univariate analysis of risk factors for CNS relapse in the entire cohort

Variable	Total (cases)	CNS relapse [cases (%)]	*P*
Stage			0.022
I or II	294	9 (3.1)	
III or IV	217	16 (7.4)	
IPI			0.026
0–2	402	16 (4.0)	
3–5	109	9 (8.3)	
Number of involved extranodal sites^a^			0.001
≤1	415	15 (3.6)	
>1	94	10 (10.6)	
Bone marrow involvement			0.043
No	493	22 (4.5)	
Yes	18	3 (16.7)	
Bone involvement			0.001
No	455	18 (4.0)	
Yes	56	7 (12.5)	
Renal involvement			<0.001
No	490	19 (3.9)	
Yes	21	6 (28.6)	
WBC count^b^			0.056
<4.0 × 10^9^/L	32	4 (12.5)	
≥4.0 × 10^9^/L	478	21 (4.4)	
LC^c^			0.027
<1.3 × 10^9^/L	169	12 (7.1)	
≥1.3 × 10^9^/L	341	13 (3.8)	
PLT^d^			0.063
<100 × 10^9^/L	21	2 (9.5)	
≥100 × 10^9^/L	489	23 (4.7)	
LDH^e^			0.015
≤1 ULN^f^	294	9 (3.1)	
>1 ULN	214	16 (7.5)	
ALP^g^			0.001
≤110 U/L	460	18 (3.9)	
>110 U/L	50	7 (14.0)	
ALB			0.005
<35 g/L	441	7 (1.6)	
≥35 g/L	70	18 (25.7)	
TP			0.063
< 60 g/L	56	7 (12.5)	
≥60 g/L	455	18 (4.0)	
HDL^h^			0.014
< 0.78 mmol/L	94	10 (10.6)	
≥0.78 mmol/L	409	14 (3.4)	
Apo-A1^i^			0.030
<1.05 g/L	205	14 (6.8)	
≥1.05 g/L	296	9 (3.0)	
Rituximab treatment			0.045
Without	135	11 (8.1)	
With	376	14 (3.7)	
IT chemotherapy prophylaxis^j^			0.053
No	446	19 (4.3)	
Yes	62	6 (9.7)	
CR with first-line therapy^k^			0.036
No	157	14 (8.9)	
Yes	349	11 (3.1)	
TTC^l^			0.003
≤108 days	261	7 (2.6)	
>108 days	222	13 (5.9)	

*WBC* white blood cell, *LC* lymphocyte count, *PLT* platelet count, *TP* total protein, *HDL* high-density lipoprotein, *Apo-A1* apolipoprotein-A1, *CR* complete remission. Other abbreviations as in Table 1

^a^The data of two patients were missing

^b^The data of one patient were missing

^c^The data of one patient were missing

^d^The data of one patient were missing

^e^The data of three patients were missing

^f^ULN for LDH: 245 U/L

^g^The data of one patient were missing

^h^The data of eight patients, including one who developed in the CNS relapse, were missing

^i^The data of ten patients, including two who developed CNS relapse, were missing

^j^The data of three patients were missing

^k^The data of five patients were missing

^l^The data of 28 patients, including five patients who developed CNS relapse, were missing

In the multivariate Cox regression analysis, bone involvement, renal involvement, ALP, ALB, treatment with rituximab, and the TTC were independently predictive of CNS relapse (Table 3).

**Table 3:** Multivariate analysis of risk factors for CNS relapse in the entire cohort

Variable	HR	95% CI	*P*
Bone involvement	4.21	1.38–12.77	0.011
Kidney involvement	3.85	1.05–14.19	0.043
ALP > 110U/L	3.59	1.25–10.34	0.018
ALB < 35g/L	3.63	1.25–10.51	0.018
Rituximab treatment	0.34	0.12–0.96	0.042
TTC ≤ 108 days	0.22	0.06–0.78	0.019

*HR* hazard ratio, *CI* confidence interval. Other abbreviations as in Table 1

### CNS relapse and its risk factors in the R-CHOP set

**Fig. 3:**
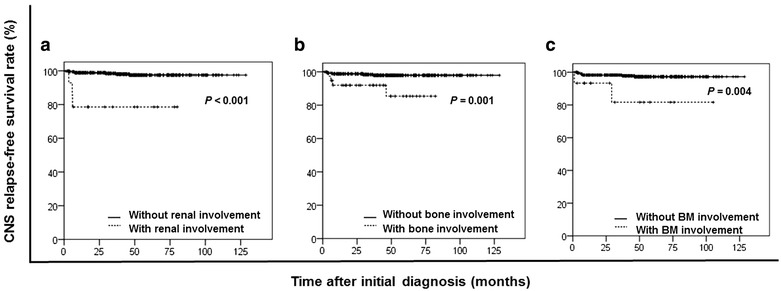
Kaplan–Meier analysis estimated CNS relapse-free survival for the 376 patients in the R-CHOP (rituximab, cyclophosphamide, doxorubicin, vincristine, and prednisone) set stratified by independent risk factors. Renal involvement (**a**), bone involvement (**b**), and bone marrow (BM) involvement (**c**) were associated with short CNS relapse-free survival

To evaluate the risk factors for CNS relapse among patients treated with R-CHOP regimen, univariate and multivariate analyses of the R-CHOP set were performed. IT chemotherapy prophylaxis still showed no protective effect against CNS relapse: the numbers of patients who experienced CNS relapse in the groups treated with and without IT chemotherapy prophylaxis were 4 (8.5%) and 11 (3.3%), and the 3-year CNS relapse rates in the IT chemotherapy prophylaxis and non-IT prophylaxis groups were 6.4% and 1.8% (*P* = 0.104). A longer TTC was no longer associated with an increased risk of CNS relapse. In both the univariate and the multivariate analyses, fewer factors were found to be significantly predictive of CNS relapse in the individual groups than in the entire cohort. Univariate analysis showed that advanced-stage disease, an IPI ≥3, involvement of more than one extranodal site, involvement of the bone marrow, bone, kidney, or testicle, an elevated ALP level (>110 U/L), and reduced HDL level (<0.78 mmol/L) were significant risk factors. Other factors with *P* values less than 0.10 were an absolute lymphocyte count <1.3 × 10^9^/L and treatment with IT chemotherapy prophylaxis. Details are listed in Table 4, and the survival curves with independent risk factors can be observed in Fig. 3.

**Table 4:** Univariate analysis of risk factors for CNS relapse in the R-CHOP set

Variable	Total (cases)	CNS relapse [cases (%)]	*P*
Stage			0.049
I or II	217	4 (1.8)	
III or IV	159	10 (6.3)	
IPI			0.017
0–2	289	7 (2.4)	
3–5	87	7 (8.0)	
Number of involved extranodal sites^a^			0.004
≤1	303	7 (2.3)	
>1	71	7 (9.9)	
Bone marrow involvement			0.004
No	360	11 (3.1)	
Yes	16	3 (18.8)	
Bone involvement			0.001
No	335	9 (2.7)	
Yes	41	5 (12.2)	
Renal involvement			<0.001
No	360	10 (2.8)	
Yes	16	4 (25.0)	
Testicular involvement			0.043
No	368	13 (3.5)	
Yes	8	1 (12.5)	
LC			0.081
<1.3 × 10^9^	132	12 (9.1)	
≥1.3 × 10^9^	244	2 (0.8)	
ALP			0.019
≤110 U/L	341	10 (2.9)	
>110 U/L	35	4 (11.4)	
HDL^b^			0.028
<0.78 mmol/L	64	10 (15.6)	
≥0.78 mmol/L	307	4 (1.3)	
IT chemotherapy prophylaxis^c^			0.083
No	329	10 (3.0)	
Yes	44	4 (9.1)	

Abbreviations as in Table 1

^a^The data of two patients were missing

^b^The data of five patients were missing

^c^The data of three patients were missing

In the multivariate analysis, involvement of the bone, bone marrow, and kidney were independent predictive factors for CNS relapse (Table 5).

**Table 5:** Multivariate analysis of risk factors for CNS relapse in the R-CHOP set

Variable	HR	95% CI	*P*
Bone involvement	5.04	1.20–21.24	0.028
Bone marrow involvement	12.63	2.39–66.74	0.003
Renal involvement	7.24	1.50–34.95	0.014

Abbreviations as in Tables 1 and 3

### OS and PFS of patients

**Fig. 4:**
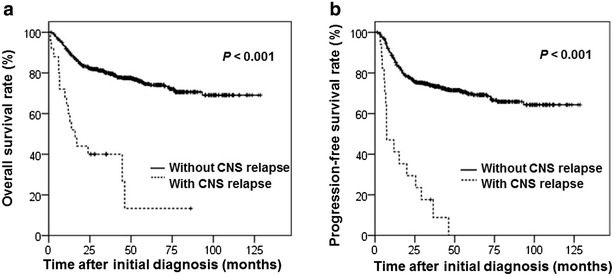
Kaplan–Meier analysis estimated overall survival (OS) and progression-free survival (PFS) for 511 DLBCL patients with or without CNS relapse. CNS relapse was associated with short OS (**a**) and short PFS (**b**)

The outcomes of patients with CNS relapse were much worse than those of patients without CNS relapse. The median OS and PFS were 16.0 and 7.3 months for the whole cohort. The 5-year OS rates in patients with or without CNS relapse were 13.3% and 74.6% (*P* < 0.001); the 5-year PFS rates in patients with or without CNS relapse were 0 and 69.7% (*P* < 0.001). Seventeen (68.0%) of the 25 patients who experienced CNS relapse died, and the median survival after CNS relapse was 8 months (range 4–16 months). The OS and PFS curves can be visualized in Fig. 4.

## Discussion

In this retrospective study of 511 newly diagnosed DLBCL cases, we demonstrated the effect of rituximab and found the current IT prophylactic strategy to be insufficient in decreasing the incidence of CNS relapse. We also established three new independent risk factors for CNS relapse: ALB level, ALP level, and the TTC. The impact of the TTC on CNS relapse indicated that the response to treatment could also be a risk factor for predicting CNS relapse in addition to the clinical characteristics at diagnosis. In the R-CHOP set, the involvement of certain specific extranodal sites (the bone, bone marrow, and kidney) as independent risk factors showed a tendency to increase CNS relapse risk.

Several studies have reported the impact of rituximab on reducing CNS relapse. In the “rituximab with CHOP over age 60 years” (RICOVER-60) trial, 1222 elderly patients with aggressive B-cell lymphoma were treated with six or eight cycles of CHOP regimen every 2 weeks with or without rituximab [20]. The 2-year CNS relapse rate was significantly lower in the R-CHOP group than in the CHOP group (4.1% vs. 6.9%, *P* = 0.043). Nonetheless, the patients in this prospective study did not all have DLBCL; other B-cell lymphomas comprised approximately 18.4% of cases. Shimazu et al. [21] examined 403 patients with DLBCL and found a trend of reduced occurrence of CNS relapse in patients treated with rituximab. However, several other studies reached different conclusions. In the Groupe d’Etude des Lymphomes de l’Adulte (GEAL) trial of 399 elderly lymphoma patients treated with 8 cycles of R-CHOP or CHOP regimen, adding rituximab showed no influence on CNS relapse [22]. This difference may be due to the varying patient characteristics in each study. In our study, rituximab treatment was found to be an independent risk factor for CNS relapse. Although the penetration of rituximab through the blood–brain barrier is extremely poor, it may still reduce CNS relapse by better systemic control of the disease [19].

As a simple approach to CNS prophylaxis, IT chemotherapy is usually administered to high-risk patients with involvement of specific extranodal sites. Recently published data suggest that CNS prophylaxis alone may not be effective in reducing CNS relapse. In the 20-year follow-up analysis by the Southwest Oncology Group (SWOG) [20], 899 patients with aggressive lymphoma were enrolled, 34 of whom had bone marrow involvement and received at least one dose of IT therapy. The study showed no significant benefit in the prevention of CNS relapse in patients who received IT chemotherapy prophylaxis [23]. A study by Tomita et al. [24] retrospectively analyzed the data of 322 patients who achieved their first CR after R-CHOP therapy and concluded that IT administration of MTX was insufficient to prevent CNS relapse. Consistent with these studies, we did not find a significantly protective effect of IT administration against CNS relapse in either the entire cohort or the R-CHOP set. For the patients with high risk of CNS relapse identified either at the discretion of the physician in our hospital or according to the NCCN model, IT chemotherapy prophylaxis showed no significant effect on preventing CNS relapse. The limited impact of IT chemotherapy prophylaxis on CNS relapse was consistent with the understanding that it is difficult to achieve therapeutic levels of MTX and Ara-C in the brain parenchyma via this route of administration [3]. A systemic treatment with high-dose MTX may be necessary in patients with high-risk features [25].

Renal involvement is uncommon in DLBCL patients. Certain studies have described patients with renal involvement as having a poor outcome due to a high rate of CNS relapse. Villa et al. [26] reported a retrospective study of 2656 DLBCL patients, among whom 52 patients had renal involvement at diagnosis. Of the 52 patients, 20 (36%) developed CNS relapse shortly after diagnosis and treatment, with a median time to relapse of 5.6 months. In our study, renal involvement was a significant risk factor for CNS relapse in univariate and multivariate analyses of both the entire cohort and the R-CHOP set.

Bone and bone marrow involvement has also been noted to carry a high risk of CNS relapse. Tomita et al. [27] examined 1221 patients with DLBCL who received R-CHOP regimen as primary therapy and noted the cumulative 5-year probability of CNS relapse to be 8.4%. Bone involvement [relative risk (RR) = 2.0, 95% CI 1.1–4.0] was found to be an independent risk factor for CNS relapse. Regarding bone marrow involvement, Shimazu et al. [21] reported a study of 403 patients with DLBCL, and the multivariate analysis identified bone marrow involvement (RR = 2.099, 95% CI 1.049–4.200) as an independent predictor of CNS relapse. Our study found that bone involvement carried a relatively high risk of CNS relapse in both the entire cohort and the R-CHOP set.

Certain studies have provided evidence that testicular lymphoma is associated with a high rate of CNS relapse. Guirguis et al. [28] reported a retrospective study of 214 DLBCL patients and found that testicular involvement was the only significant prognostic factor for CNS relapse. Park et al. [29] performed an analysis of 45 patients with primary testicular lymphoma, among whom 20% experienced CNS relapse, with a median follow-up duration of 31.6 months. Many hospitals as well as our cancer center routinely administer IT chemotherapy to patients with testicular involvement. However, testicular involvement was a high risk factor only in the univariate analysis of the R-CHOP set in our study. As testicular involvement was only detected in 12 of 511 patients, this result may be attributable to the small number of specific patients.

Hollender et al. [30] built a risk model for CNS relapse in patients with NHL. A total of 2514 patients were enrolled, and 1220 were found to have high-grade histology. Among these high-grade B-cell and T-cell lymphoma patients, five independent risk factors for CNS relapse were identified: elevated LDH level, serum ALB <35 g/L, <60 years of age, retroperitoneal lymph node involvement, and involvement of more than one extranodal site. Serum ALB <35 g/L was also reported as a risk factor in the risk model for CNS relapse of high-grade lymphoma. In our study of the entire cohort, the patients with ALB <35 g/L (HR = 3.63, 95% CI 1.25–10.51) showed a relatively high risk for CNS relapse, and ALB <35 g/L was identified as an independent risk factor. This finding suggests that serum ALB level is a risk factor for CNS relapse when the analysis is restricted to DLBCL patients. ALP is a type of plasma membrane-bound glycoprotein that is widely distributed in human tissue, and its elevation in the serum indicates the presence of involvement of the bone, liver, and other sites [31]. In our study, elevated ALP level (HR = 3.59, 95% CI 1.25–10.34) was found to increase the rate of CNS relapse in the univariate and multivariate analyses. The mechanism of this risk factor should be further explored.

In our study, the DLBCL patients who failed to achieve CR quickly had a high risk of CNS relapse. Considering this finding, salvage chemotherapy of CNS prophylaxis may be necessary for patients with late CR and for those who fail to achieve CR. Compared with the entire cohort, there were fewer risk factors for CNS relapse in the R-CHOP set. The use of rituximab may decrease the risk level of many risk factors for CNS relapse. Among those treated with R-CHOP regimen, efforts to counteract CNS relapse should be focused on patients with involvement of specific extranodal sites.

In conclusion, CNS relapse is an uncommon but fatal event in DLBCL patients. Rituximab appears to decrease the rate of CNS relapse, and the effect of IT chemotherapy prophylaxis seems limited. ALB level, ALP level, and the TTC are new risk factors for predicting CNS relapse. In patients treated with R-CHOP regimen, there is a trend of increased risk for those with involvement of specific extranodal sites, such as the kidney, bone, and bone marrow. For patients with a high risk of CNS relapse, prophylactic strategies, such as systemic high-dose MTX, should be recommended.

## References

[1] Martelli M, Ferreri AJM, Agostinelli C, Di Rocco A, Pfreundschuh M, Pileri SA (2013). Diffuse large B-cell lymphoma. Crit Rev Oncol Hematol.

[2] Roschewski M, Staudt L, Wilson W (2014). Diffuse large B-cell lymphoma treatment approaches in the molecular era. Nat Rev Clin Oncol.

[3] Zhang J, Chen B, Xu X (2014). Impact of rituximab on incidence of and risk factors for central nervous system relapse in patients with diffuse large B-cell lymphoma: a systematic review and meta-analysis. Leuk Lymphoma.

[4] Fletcher CD, Kahl BS (2014). Central nervous system involvement in diffuse large B-cell lymphoma: an analysis of risks and prevention strategies in the post-rituximab era. Leuk Lymphoma.

[5] Chihara D, Oki Y, Matsuo K, Onoda H, Taji H, Yamamoto K (2011). Incidence and risk factors for central nervous system relapse in patients with diffuse large B-cell lymphoma: analyses with competing risk regression model. Leuk Lymphoma.

[6] Tai WM, Chung J, Tang PL, Koo YX, Hou X, Tay KW (2011). Central nervous system (CNS) relapse in diffuse large B cell lymphoma (DLBCL): pre- and post-rituximab. Ann Hematol.

[7] Savage KJ, Johnson NA, Ben-Neriah S, Connors JM, Sehn LH, Farinha P (2009). MYC gene rearrangements are associated with a poor prognosis in diffuse large B-cell lymphoma patients treated with R-CHOP chemotherapy. Blood.

[8] Rowe M, Fitzsimmons L, Bell AI (2014). Epstein–Barr virus and Burkitt lymphoma. Chin J Cancer.

[9] Siegal T, Goldschmidt N (2012). CNS prophylaxis in diffuse large B-cell lymphoma: if, when, how and for whom?. Blood Rev.

[10] Coiffier B, Lepage E, Briere J, Herbrecht R, Tilly H, Bouabdallah R (2002). CHOP chemotherapy plus rituximab compared with CHOP alone in elderly patients with diffuse large-B-cell lymphoma. N Engl J Med.

[11] Feugier P, Van Hoof A, Sebban C, Solal-Celigny P, Bouabdallah R, Fermé C (2005). Long-term results of the R-CHOP study in the treatment of elderly patients with diffuse large B-cell lymphoma: a study by the Groupe d’Etude des Lymphomes de l’Adulte. J Clin Oncol.

[12] Habermann TM, Weller EA, Morrison VA, Gascoyne RD, Cassileth PA, Cohn JB (2006). Rituximab-CHOP versus CHOP alone or with maintenance rituximab in older patients with diffuse large B-cell lymphoma. J Clin Oncol.

[13] Pfreundshcuh M, Trümper L, Österborg A, Pettengell R, Trneny M, Imrie K (2006). CHOP-like chemotherapy plus rituximab versus CHOP-like chemotherapy alone in young patients with good prognosis diffuse large B-cell lymphoma: a randomised controlled trial by the MabThera International Trial (MInT) Group. Lancet Oncol.

[14] He XH, Li B, Yang S, Lu N, Zhang X, Zou SM (2012). R-CHOP regimen can significantly decrease the risk of disease relapse and progression in patients with non-germinal center B-cell subtype diffuse large B-cell lymphoma. Chin J Cancer.

[15] Shi Y, Zhou P, Han X, He X, Zhou S, Liu P (2015). Autologous peripheral blood stem cell mobilization following dose-adjusted cyclophosphamide, doxorubicin, vincristine, and prednisolone chemotherapy alone or in combination with rituximab in treating high-risk non-Hodgkin’s lymphoma. Chin J Cancer.

[16] Chen KL, Chen J, Rao HL, Guo Y, Huang HQ, Zhang L (2015). Hepatitis B virus reactivation and hepatitis in diffuse large B-cell lymphoma patients with resolved hepatitis B receiving rituximab-containing chemotherapy: risk factors and survival. Chin J Cancer.

[17] Law MF, Chan HN, Lai HK, Ha CY, Ng C, Yeung YM (2015). Effects of addition of rituximab to chemotherapy on central nervous system events in patients with diffuse large B-cell lymphoma. Mol Clin Oncol.

[18] Ferreri AJM, Bruno-Ventre M, Donadoni G, Ponzoni M, Citterio G, Foppoli M (2015). Risk-tailored CNS prophylaxis in a mono-institutional series of 200 patients with diffuse large B-cell lymphoma treated in the rituximab era. Br J Haematol.

[19] Villa D, Connors JM, Shenkier TN, Gascoyne RD, Sehn LH, Savage KJ (2010). Incidence and risk factors for central nervous system relapse in patients with diffuse large B-cell lymphoma: the impact of the addition of rituximab to CHOP chemotherapy. Ann Oncol.

[20] Boehme V, Schmitz N, Zeynalova S, Loeffler M, Pfreundschuh M (2009). CNS events in elderly patients with aggressive lymphoma treated with modern chemotherapy (CHOP-14) with or without rituximab: an analysis of patients treated in the RICOVER-60 trial of the German High-Grade Non-Hodgkin Lymphoma Study Group (DSHNHL). Blood.

[21] Shimazu Y, Notohara K, Ueda Y (2009). Diffuse large B-cell lymphoma with central nervous system relapse: prognosis and risk factors according to retrospective analysis from a single-center experience. Int J Hematol.

[22] Feugier P, Virion J, Tilly H, Haioun C, Marit G, Macro M (2004). Incidence and risk factors for central nervous system occurrence in elderly patients with diffuse large-B-cell lymphoma: influence of rituximab. Ann Oncol.

[23] Bernstein S, Unger J, Leblanc M, Friedberg J, Miller T, Fisher R (2009). Natural history of CNS relapse in patients with aggressive non-Hodgkin’s lymphoma: a 20-year follow-up analysis of SWOG 8516—the Southwest Oncology Group. J Clin Oncol.

[24] Tomita N, Takasaki H, Ishiyama Y, Kishimoto K, Ishibashi D, Koyama S (2015). Intrathecal methotrexate prophylaxis and central nervous system relapse in patients with diffuse large B-cell lymphoma following rituximab plus cyclophosphamide, doxorubicin, vincristine and prednisone. Leuk Lymphoma.

[25] Cheah CY, Herbert KE, O’Rourke K, Kennedy GA, George A, Fedele PL (2014). A multicentre retrospective comparison of central nervous system prophylaxis strategies among patients with high-risk diffuse large B-cell lymphoma. Br J Cancer.

[26] Villa D, Connors JM, Sehn LH, Gascoyne RD, Savage KJ (2011). Diffuse large B-cell lymphoma with involvement of the kidney: outcome and risk of central nervous system relapse. Haematologica.

[27] Tomita N, Yokoyama M, Yamamoto W, Watanabe R, Shimazu Y, Masaki Y (2012). Central nervous system event in patients with diffuse large B-cell lymphoma in the rituximab era. Cancer Sci.

[28] Guirguis HR, Cheung MC, Mahrous M, Piliotis E, Berinstein N, Imrie KR (2012). Impact of central nervous system (CNS) prophylaxis on the incidence and risk factors for CNS relapse in patients with diffuse large B-cell lymphoma treated in the rituximab era: a single centre experience and review of the literature. Br J Haematol.

[29] Park B, Kim JG, Sohn SK, Kang HJ, Lee SS, Eom HS (2007). Consideration of aggressive therapeutic strategies for primary testicular lymphoma. Am J Hematol.

[30] Hollender A, Kvaloy S, Nome O, Skovlund E, Lote K, Holte H (2002). Central nervous system involvement following diagnosis of non-Hodgkin’s lymphoma: a risk model. Ann Oncol.

[31] Sharma U, Pal D, Prasad R (2013). Alkaline phosphatase: an overview. Indian J Clin Biochem.

